# Arsenic Exposure and Hypertension: A Systematic Review

**DOI:** 10.1289/ehp.1103988

**Published:** 2011-12-02

**Authors:** Lalita N. Abhyankar, Miranda R. Jones, Eliseo Guallar, Ana Navas-Acien

**Affiliations:** 1 Department of Environmental Health Sciences, and; 2 Department of Epidemiology and Welch Center for Prevention, Epidemiology and Clinical Research, Johns Hopkins Bloomberg School of Public Health, Baltimore, Maryland, USA; 3 Department of Cardiovascular Epidemiology and Population Genetics, Centro Nacional de Investigaciones Cardiovasculares, Madrid, Spain; 4 Department of Medicine, Johns Hopkins Medical Institutions, Baltimore, Maryland, USA

**Keywords:** arsenic, blood pressure, hypertension, meta-analysis, systematic review

## Abstract

Background: Environmental exposure to arsenic has been linked to hypertension in persons living in arsenic-endemic areas.

Objective: We summarized published epidemiologic studies concerning arsenic exposure and hypertension or blood pressure (BP) measurements to evaluate the potential relationship.

Data sources and extraction: We searched PubMed, Embase, and TOXLINE and applied predetermined exclusion criteria. We identified 11 cross-sectional studies from which we abstracted or derived measures of association and calculated pooled odds ratios (ORs) using inverse-variance weighted random-effects models.

Data synthesis: The pooled OR for hypertension comparing the highest and lowest arsenic exposure categories was 1.27 [95% confidence interval (CI): 1.09, 1.47; *p*-value for heterogeneity = 0.001; *I*^2^ = 70.2%]. In populations with moderate to high arsenic concentrations in drinking water, the pooled OR was 1.15 (95% CI: 0.96, 1.37; *p*-value for heterogeneity = 0.002; *I*^2^ = 76.6%) and 2.57 (95% CI: 1.56, 4.24; *p*-value for heterogeneity = 0.13; *I*^2^ = 46.6%) before and after excluding an influential study, respectively. The corresponding pooled OR in populations with low arsenic concentrations in drinking water was 1.56 (95% CI: 1.21, 2.01; *p*-value for heterogeneity = 0.27; *I*^2^ = 24.6%). A dose–response assessment including six studies with available data showed an increasing trend in the odds of hypertension with increasing arsenic exposure. Few studies have evaluated changes in systolic and diastolic BP (SBP and DBP, respectively) measurements by arsenic exposure levels, and those studies reported inconclusive findings.

Conclusion: In this systematic review we identified an association between arsenic and the prevalence of hypertension. Interpreting a causal effect of environmental arsenic on hypertension is limited by the small number of studies, the presence of influential studies, and the absence of prospective evidence. Additional evidence is needed to evaluate the dose–response relationship between environmental arsenic exposure and hypertension.

Hypertension is a major risk factor for mortality and morbidity worldwide (Lopez et al. 2006; Murray and Lopez 1997; Oparil et al. 2003; Whitworth 2003). Risk factors for hypertension include high salt intake, increased body mass index (BMI), genetic predisposition, and exposure to psychosocial stress (Oparil et al. 2003; Whitworth 2003). Additional evidence, however, suggests that environmental factors play a role in hypertension development (Houston 2007; Klahr 2001; Laclaustra et al. 2009; Navas-Acien et al. 2007, 2008; Oparil et al. 2003; Tellez-Plaza et al. 2008; Vaziri 2008). The identification and mitigation of environmental exposures related to hypertension could contribute to reducing the worldwide burden of hypertension-related disease.

Among environmental exposures, epidemiologic and experimental evidence supports the possibility that arsenic plays a role in hypertension and other cardiometabolic diseases [Chen Y et al. 2011; Medrano et al. 2010; Navas-Acien et al. 2005; Smedley and Kinniburgh 2002; U.S. Department of Health and Human Services (DHHS) 2005; Wang CH et al. 2007; Wu et al. 1989]. Arsenic-contaminated drinking water represents a major public health problem internationally (Chappell et al. 2002; Chen CJ et al. 1995; Chilvers and Peterson 1987; Hinkle and Polette 1999; Mukherjee et al. 2006; Rahman et al. 1999). The World Health Organization and U.S. Environmental Protection Agency (EPA) standard for arsenic levels in drinking water is 10 μg/L (DHHS 2005; Whitworth 2003). In the United States alone, millions of persons are exposed to arsenic concentrations > 10 μg/L; whereas persons in Bangladesh, China, India, Cambodia, Ghana, Argentina, Mexico, and other countries around the world are exposed to arsenic levels in drinking water that are well beyond 10 μg/L (Navas-Acien et al. 2005; DHHS 2005). Epidemiologic studies conducted in arsenic-endemic areas in Taiwan and Bangladesh have found a positive relationship between inorganic arsenic exposure from drinking water and hypertension (Chen CJ et al. 1995; Rahman et al. 1999). Experimental studies have indicated that arsenic exposure may be involved in the development of hypertension through the promotion of inflammation, oxidative stress, and endothelial dysfunction (Aposhian et al. 2003; Balakumar et al. 2008; Lee et al. 2003; Smedley and Kinniburgh 2002; DHHS 2005).

To evaluate the potential relationship between arsenic and hypertension, we conducted a systematic review of epidemiologic studies that have investigated the association between inorganic arsenic exposure (using environmental measures or biomarkers) and hypertension outcomes [using hypertension status and systolic and diastolic blood pressure (SBP and DBP, respectively)].

## Methods

**Figure 1:**
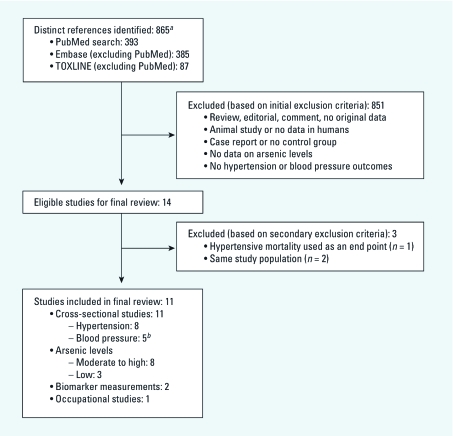
Summary of search and screening process. ***^a^***A total of 138 studies were not in English. ***^b^***Chen Y et al. (2007) and Jones et al. (2011) were the only studies including both hypertension and BP level end points.

*Search strategy and data abstraction.* We searched PubMed (http://www.ncbi.nlm.nih.gov/pubmed/, Embase (http://www.embase.com/home), and TOXLINE (http://toxnet.nlm.nih.gov/cgi-bin/sis/htmlgen?TOXLINE) databases to find all published observational studies evaluating the relationship between arsenic exposure with hypertension or BP levels using the free text and Medical Subject Headings (MeSH) terms “arsenic,” “arsenicals,” “arsenate,” or “arsenite” and “hypertension” or “blood pressure.” The search period was January 1966 through March 2011 with no language restrictions (Figure 1).

Two investigators (L.N.A. and M.R.J.) reviewed each paper and applied the study selection criteria (Figure 1). Epidemiologic studies with data on arsenic exposure and hypertension outcomes were included. We excluded nonoriginal reports, experimental studies, case reports and case series, and studies without measures of arsenic exposure or hypertension end points. We also excluded one study that used hypertension mortality as the only end point (Lewis et al. 1999) and two reports (Hsueh et al. 2005; Huang et al. 2007) that used the same study population as another included study (Chen CJ et al. 1995). The two investigators, L.N.A. and M.R.J., independently abstracted the study data, including design, study population (location, age, and sex distribution), sample size, arsenic assessment and exposure levels, hypertension outcomes, study results (measures of association), and potential confounders accounted for in the statistical analysis. Authors were contacted for information unavailable in the published reports. For studies with multiple levels of adjustment, we abstracted the measure of association obtained from the model adjusted for the most covariates after confirmation that adjustment did not markedly modify the conclusions of any individual study. For studies that were not in English (138 of 865), the full text of the article was translated by a native speaker if the information in the abstract was insufficient to include/exclude the article. Discrepancies were resolved by consensus. The quality of the included studies was evaluated by adapting the criteria developed by Longnecker et al. (1988) and Appel et al. (2002).

*Statistical analysis.* For studies that reported hypertension, we abstracted (Chen CJ et al. 1995; Chen Y 2007; Jones et al. 2011; Rahman et al. 1999; Zierold et al. 2004) or derived (Guo et al. 2007; Wang SL et al. 2007; Yildiz et al. 2008) odds ratios (ORs) and prevalence ratios for hypertension and their standard errors from the published data. For three studies with hypertension data but no available measures of association, we estimated the OR and 95% confidence interval (CI) for hypertension by arsenic categories using the number of cases and noncases in the exposed and unexposed groups (Guo et al. 2007; Wang SL et al. 2007; Yildiz et al. 2008). For summary purposes, we pooled OR estimates comparing hypertension in the highest and lowest categories of arsenic exposure from individual studies using an inverse-variance weighted random-effects model. Pooled ORs were calculated for all studies and separately for studies conducted in populations exposed to moderate-to-high arsenic levels and for studies conducted in populations exposed to low arsenic levels. Heterogeneity was quantified with the *I*^2^ statistic, an index that describes the proportion of the total variation in pooled estimates due to heterogeneity (Higgins and Thompson 2002). The relative influence of each study on pooled estimates was estimated by omitting one study at a time. Finally, we assessed publication bias using funnel plots. For studies that reported hypertension results for three or more arsenic categories, we evaluated the dose–response relationship over the range of arsenic levels (Chen CJ et al. 1995; Jones et al. 2011; Rahman et al. 1999; Wang SL et al. 2007; Zierold et al. 2004). All statistical analyses were performed using Stata software, version 11.0 (StataCorp, College Station, TX, USA).

For studies that reported SBP (Chen Y et al. 2007; Dastgiri et al. 2010; Jensen and Hansen 1998; Jones et al. 2011; Kwok et al. 2007) and DBP (Chen Y et al. 2007; Dastgiri et al. 2010; Jones et al. 2011; Kwok et al. 2007) levels, we abstracted (Jones et al. 2011; Kwok et al. 2007) or derived (Chen Y et al. 2007; Dastgiri et al. 2010; Jensen and Hansen 1998) the difference in BP levels comparing the highest and lowest categories of arsenic exposure. Because the number of studies was small and because the largest study (Chen Y et al. 2007) did not provide enough information to calculate CIs, these results are presented descriptively, and no pooled estimate was calculated.

## Results

*Study characteristics.* Eleven studies, published between 1995 and 2011, were identified (Table 1). All studies meeting the inclusion criteria were cross-sectional and published in English. Combined, the studies covered arsenic exposure and hypertension outcomes for > 20,000 individuals. Eight studies were conducted at moderate to high levels of exposure (average levels in drinking water ≥ 50 μg/L or occupational studies) (Chen CJ et al. 1995; Chen Y 2007; Dastgiri et al. 2010; Guo et al. 2007; Jensen and Hansen 1998; Kwok et al. 2007; Rahman et al. 1999; Yildiz et al. 2008), and three studies were conducted at low levels of exposure (average levels in drinking water < 50 μg/L) (Jones et al. 2011; Wang SL et al. 2007; Zierold et al. 2004). Ten studies were conducted in general populations (two from Taiwan, two from Bangladesh, two from Inner Mongolia, two from the United States, one from Turkey, and one from Iran) (Chen CJ et al. 1995; Chen Y 2007; Dastgiri et al. 2010; Guo et al. 2007; Jones et al. 2011; Kwok et al. 2007; Rahman et al. 1999; Wang SL et al. 2007; Yildiz et al. 2008; Zierold et al. 2004). One study was conducted in an occupational setting in Denmark (Jensen and Hansen 1998). Five studies measured arsenic concentrations in drinking water (Chen CJ et al. 1995; Chen Y 2007; Kwok et al. 2007; Rahman et al. 1999; Zierold et al. 2004), three compared areas of high and low arsenic concentrations in drinking water (Dastgiri et al. 2010; Guo et al. 2007; Yildiz et al. 2008), two studies used biomarkers (hair, Wang SL et al. 2007; urine, Jones et al. 2011), and one study assigned arsenic exposure based on job title (Jensen and Hansen 1998). Eight studies assessed hypertension as the end point of interest (Chen CJ et al. 1995; Chen Y 2007; Guo et al. 2007; Jones et al. 2011; Rahman et al. 1999; Wang SL et al. 2007; Yildiz et al. 2008; Zierold et al. 2004), five studies reported differences in mean SBP (Chen Y et al. 2007; Dastgiri et al. 2010; Jensen and Hansen 1998; Jones et al. 2011; Kwok et al. 2007), and four studies reported differences in mean DBP (Chen Y et al. 2007; Dastgiri et al. 2010; Jones et al. 2011; Kwok et al. 2007).

**Table 1:** Epidemiological studies of arsenic exposure and blood pressure end points.

Reference	*n*	Percent men	Arsenic			Definition of hypertension	No. of cases	Adjustment variables
			Country	Population	Age				Marker	Mean ± SD	Range	SBP/DBP determinations
Moderate to high arsenic levels in drinking water (average ≥ 50 μg/L) or occupationally exposed populations														
Chen CJ et al. 1995	Southwest Taiwan	General	898	42.5	≥ 30 years		CAE in groundwater	NR	0 to > 18.5 mg/L-years		Mean of three SBP and DBP measures after 20 min of rest with mercury sphygmomanometer	SBP ≥ 160 mmHg, DBP ≥ 95 mmHg, HT medication	168	Age, sex, BMI, diabetes, proteinuria, fasting serum triglycerides
Jensen and Hansen 1998	Denmark	Occupational	59	NR	Mean age, 37 years		Occupational exposure (confirmed in urine)	Exposed,*a* 14.8 μg /g creatinine Unexposed, 7.9 μg /g	Exposed,*a* 7.6–195.6 Unexposed, 3.9–29.1		Mean of three SBP and DBP measures after 10 min of rest with digital equipment	NA	NA	None
Rahman et al. 1999	Central and eastern Bangladesh	General	1,595	59.7	30–85 years		CAE in groundwater	NR	0 to > 10 mg/L-years		Lowest BP of three measures used; two additional measurements taken for individuals w/HT	SBP ≥ 140 mmHg and/or DBP ≥ 90 mmHg	207	Age, sex, BMI
Chen Y et al. 2007	Araihazar, Bangladesh	General	11,458	42.8	≥ 18 years		TWA concentration in groundwater	NR	0.1–864.0 μg/L		SBP and DBP measured by trained clinicians with automatic sphygmomanometer after 2–3 min of rest Two or more measures taken for persons with SBP/DBP ≥ 140/90 mmHg at first measure Lowest BP used	SBP ≥ 140 mmHg and/or DBP ≥ 90 mmHg	1,360	Age, sex, BMI, smoking, education, daily water consumption
Guo et al. 2007	Inner Mongolia, China	General	869	NA	Childbearing age		High vs. low arsenic in water	NR	50–1,860 μg/L		NA	NR	56	None
Kwok et al. 2007	Inner Mongolia, China	General (postpartum)	3,260	0.0	17–45 years		Individual groundwater concentration	NR	< LOD*a* to > 100 μg/L		SBP and DBP measured after 5 min of rest at 6 weeks postpartum using appropriately sized cuff	NA	NA	Age, body weight
Yildiz et al. 2008	Dulkadir and Alikoy, Turkey	General	80	100	Mean age, 35 years		High vs. low arsenic in water	659 ± 323 μg/L	422–1,066 μg/L		NR	NR	14	None
Dastgiri et al. 2010	Ghopuz and Mayan, Iran	General	208	42.7	≥ 6 years; mean age, 33 years		High vs. low arsenic in water	1.031 mg/L	NR		SBP and DBP measured once after 10 min rest using portable sphygmomanometer	NA	NA	None
Low arsenic levels in drinking water (average < 50 μg/L)														
Zierold et al. 2004	Wisconsin, USA	General	1,185	NA	≥ 35 years		Individual groundwater concentration	Median, 2 μg/L	0–2,389 μg/L		NA	Self-reported	NR	Age, sex, BMI, smoking
Wang SL et al. 2007	Central Taiwan	General	432	44.2	35–64 years		Hair, total arsenic	0.071 μg/g creatinine	NR		Mean of two SBP and DBP measures using mercury sphygmomanometer with appropriately sized cuff Two measures carried out 30 min apart; if difference > 5%, BP measured third time and two closest used	SBP ≥ 140 mmHg and/or DBP ≥ 90 mmHg, HT medication	NR	None
Jones et al. 2011	USA	General	4,167	49.0	Mean age, 47.7 years		Urine arsenic (μg/L)	Median, 8.3 μg/L	< 0.6 to > 17.1 μg/L		Mean of three or four SBP and DBP measures by certified examiners using appropriately sized cuff after 5 min rest	SBP ≥ 140 mmHg and/or DBP ≥ 90 mmHg, HT medication	1,761	Age, sex, race, ethnicity, urine creatinine, education, BMI, serum cotinine, arsenobetaine
Abbreviations: BP, blood pressure; CAE, cumulative arsenic exposure, assessed by measuring the arsenic concentration in groundwater at the village level multiplied by the drinking duration at the individual level (Chen CJ et al. 1995); HT, hypertension; LOD, limit of detection; NA, not available; NR, not reported; TWA, time-weighted arsenic concentration, calculated as Σ*CiTi*/Σ*Ti*, where “*Ci* and *Ti* denote the well arsenic concentration and drinking duration for the *i*th well” (Chen Y et al. 2007). **a**Not used in the statistical analysis; reported exclusively to confirm arsenic differences in exposed and unexposed participants.														

*Quality assessment.* Five studies measured arsenic in drinking water at the individual level (Chen Y et al. 2007; Jones et al. 2011; Kwok et al. 2007; Wang SL et al. 2007; Yildiz et al. 2008); three of these studies measured individual arsenic exposure based on measured well water concentrations (Chen Y et al. 2007; Kwok et al. 2007; Yildiz et al. 2008), and two studies used a biomarker of exposure (Table 2) (Jones et al. 2011; Wang SL et al. 2007). Five studies defined hypertension based on established cutoffs for SBP and DBP levels measured with a standardized protocol and self-reported physician diagnosis or antihypertensive treatment (Chen CJ et al. 1995; Chen Y 2007; Jones et al. 2011; Rahman et al. 1999; Wang SL et al. 2007). Five of the 11 studies did not adjust for potential confounders (Dastgiri et al. 2010; Guo et al. 2007; Jensen and Hansen 1998; Wang SL et al. 2007; Yildiz et al. 2008). Other studies adjusted at least for age, sex, and BMI.

**Table 2:** Criteria for evaluation of design and data analysis of epidemiological studies on arsenic and hypertension.*^a^*

Moderate to high arsenic levels (average ≥ 50 μg/L)*b*															Low arsenic levels (average < 50 μg/L)*c*				
Criteria	Chen CJ et al. 1995	Jensen and Hansen 1998	Rahman et al. 1999	Chen Y et al. 2007	Guo et al. 2007	Kwok et al. 2007	Yildiz et al. 2008	Dastgiri et al. 2010	Zierold et al. 2004	Wang SL et al. 2007	Jones et al. 2011
BP was measured on participant in a seated position, using multiple SDP and DBP measures on the same arm with an appropriately sized cuff and after several minutes of rest*d*		Yes		Yes		Yes		Yes		No		Yes		No		No		No		Yes		Yes
Standardized hypertension definition		Yes		—		Yes		Yes		No		—		No		—		No		Yes		Yes
Arsenic exposure assessed using a biomarker		No		No		No		No		No		No		No		No		No		Yes		Yes
Arsenic exposure assessed at the individual level		No		No		No		Yes		No		Yes		No		No		Yes		Yes		Yes
Response rate at least 70%		Yes		No		Yes		Yes		No		No		No		Yes		No		No		Yes
Interviewer was blinded with respect to the participant case or exposure status		Yes		No		No		Yes		No		Yes		No		No		Yes		Yes		Yes
Same exclusion criteria applied to all participants		Yes		No		Yes		Yes		Yes		Yes		Yes		Yes		Yes		Yes		Yes
Data collected in a similar manner for all participants		Yes		Yes		Yes		Yes		Yes		Yes		Yes		Yes		Yes		Yes		Yes
Noncases would have been cases had they developed hypertension		Yes		—		Yes		Yes		No		—		No		—		No		Yes		Yes
Authors controlled for relevant confounding factors in addition to age, sex, and BMI		Yes		No		Yes		Yes		No		Yes		No		No		Yes		No		Yes
—, Not applicable. **a**Criteria modified from Longnecker et al. (1988) and Appel et al. (2002). bArsenic exposure via drinking water or occupation. cArsenic exposure via drinking water only. dStudies indicating that they used the WHO protocol were considered to meet the criteria for blood pressure measurement.																						

**Figure 2:**
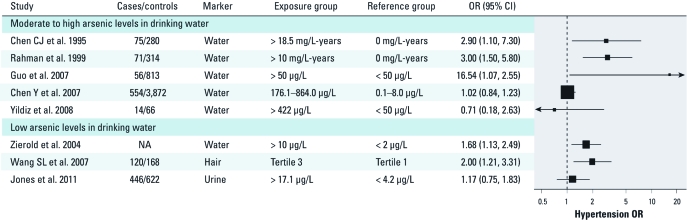
ORs of hypertension by arsenic exposure levels. The area of each square is proportional to the inverse of the variance of the estimated log OR. Horizontal lines represent 95% CIs. In the Chen Y et al. (2007) study, arsenic concentrations in drinking water were estimated based on time-weighted arsenic concentrations (Σ*C_i_T_i _*/Σ*T_i_*, where “*C_i_* and *T_i_* denote the well arsenic concentration and drinking duration for the *i*th well”).

*ORs estimates for hypertension.* For the association of hypertension with arsenic exposure, five of the eight studies found a positive association (Chen CJ et al. 1995; Guo et al. 2007; Rahman et al. 1999; Wang SL et al. 2007; Zierold et al. 2004). Among the studies that assessed hypertension at moderate to high levels of exposure, the OR estimates comparing highest with lowest arsenic exposure groups ranged from 0.71 (95% CI: 0.18, 2.63) in a small study in Turkey (Yildiz et al. 2008) to 16.5 (95% CI: 2.8, 668.5) in a study in Inner Mongolia (Figure 2) (Guo et al. 2007). The two studies from Bangladesh provided inconsistent results: an OR of 3.0 (95% CI: 1.5, 5.8) in the study by Rahman et al. (1999) and an OR of 1.02 (95% CI: 0.84, 1.23) in the study by Chen Y et al. (2007). Among the studies that assessed hypertension at low levels of exposure, the OR estimates comparing highest with lowest arsenic exposure groups ranged from 1.17 (95% CI: 0.75, 1.83) in a study in the general U.S. population (Jones et al. 2011) to 2.00 (95% CI: 1.21, 3.31) in a study in central Taiwan (Wang SL et al. 2007).

The pooled OR of hypertension comparing the highest and lowest arsenic exposure categories in the eight studies with available information on hypertension was 1.27 (95% CI: 1.09, 1.47; *p*-value for heterogeneity = 0.001; *I*^2^ = 70.2%). The corresponding pooled OR in the five studies with moderate to high arsenic exposure was 1.15 (95% CI: 0.96, 1.37; *p*-value for heterogeneity = 0.002; *I*^2^ = 76.6%), with the study by Chen Y et al. (2007) being highly influential. Excluding that study, the pooled OR was 2.57 (95% CI: 1.56, 4.24; *p*-value for heterogeneity = 0.13; *I*^2^ = 46.6%). The pooled OR comparing the highest and lowest arsenic exposure categories in the three studies with low arsenic exposure was 1.56 (95% CI: 1.21, 2.01; *p*-value for heterogeneity = 0.27; *I*^2^ = 24.6%). We also restricted the overall pooled analysis to studies with multivariable adjusted ORs (pooled OR = 1.22; 95% CI: 1.04, 1.42) (Chen CJ et al. 1995; Chen Y 2007; Jones et al. 2011; Rahman et al. 1999; Zierold et al. 2004), studies with a standard hypertension definition (pooled OR = 1.21; 95% CI: 1.03, 1.42) (Chen CJ et al. 1995; Chen Y 2007; Jones et al. 2011; Rahman et al. 1999; Wang SL et al. 2007), and studies with individual assessment of arsenic exposure (pooled OR = 1.19; 95% CI: 1.02, 1.38) (Chen Y et al. 2007; Jones et al. 2011; Wang SL et al. 2007; Zierold et al. 2004). Funnel plots did not suggest the presence of publication or related biases (data not shown).

**Figure 3:**
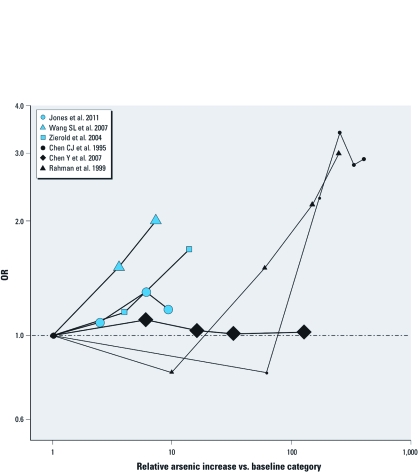
Evaluation of dose response for arsenic exposure and hypertension. Blue symbols indicate studies conducted in populations with low arsenic levels in drinking water (average < 50 μg/L); black symbols indicate studies conducted in populations with moderate-to-high arsenic levels in drinking water (average > 50 μg/L). The size of each data point is inversely weighted based on the inverse of the variance of the estimated log OR. For the Wang SL et al. (2007) study, actual arsenic levels for each hair tertile were not provided, and values defining the arsenic exposure tertiles were approximated based on the geometric mean of hair arsenic.

We evaluated the dose response for six studies with ORs reported for three or more categories (Figure 3) (Chen CJ et al. 1995; Chen Y 2007; Jones et al. 2011; Rahman et al. 1999; Wang SL et al. 2007; Zierold et al. 2004). Among them, the Chen Y et al. (2007) study in Bangladesh showed no dose–response relationship. Compared with the baseline category, the other study from Bangladesh (Rahman et al. 1999) and the study from Taiwan (Chen CJ et al. 1995) showed increased prevalence of hypertension for most of the arsenic exposure categories. Studies conducted at low levels of exposure in drinking water (Jones et al. 2011; Wang SL et al. 2007; Zierold et al. 2004) showed an increased prevalence of hypertension throughout the range of arsenic exposure levels, although the association was not statistically significant for the intermediate arsenic categories.

**Figure 4:**
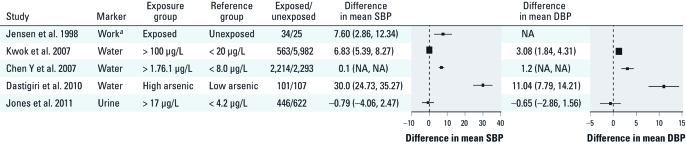
Difference (95% CI) in mean SBP and DBP by arsenic exposure level. The area of each square is proportional to the inverse of the variance of the estimated. NA: not available [the study by Chen Y et al. (2007) did not include standard errors or data that would allow estimation of the standard errors for mean systolic and diastolic blood pressure SBP and DBP levels, and a 95% CI could not be calculated for this study]. *^a^*Professions include taxidermists, garden fence makers, weekend cottage constructors, wood impregnators, electric pylon impregnators, and new house constructors (Jensen and Hansen 1998).

*Difference in BP level estimates.* For the association of arsenic exposure with BP levels, three of five studies found a positive association with SBP (Dastgiri et al. 2010; Jensen and Hansen 1998; Kwok et al. 2007), and two of four studies found a positive association with DBP (Figure 4) (Dastgiri et al. 2010; Kwok et al. 2007). The difference in BP levels comparing the highest and lowest arsenic exposure categories ranged from –0.79 to 30.0 mmHg for SBP and from –0.65 to 11.04 mmHg for DBP. Only two studies adjusted for hypertension risk factors (Jones et al. 2011; Kwok et al. 2007).

## Discussion

This systematic review identified an association between arsenic exposure and the prevalence of hypertension. The association was present both in studies conducted in areas with moderate-to-high arsenic exposure levels and in studies conducted in areas with low exposure levels. A clear dose–response was observed in several studies, and experimental evidence supports the hypertensive effects of arsenic. The interpretation of this association regarding the causal effect of arsenic on hypertension, however, is limited by the small number of studies, the heterogeneity across studies, and the absence of prospective evidence. In addition, some studies were affected by additional methodological limitations such as the lack of standard hypertension definitions, individual assessment of arsenic exposure, or appropriate adjustment for relevant confounders. The evidence is particularly scarce for low levels of exposure and for evaluating the association with SBP and DBP levels as continuous outcomes. Overall, the evidence is suggestive but insufficient to infer a causal relationship between environmental arsenic exposure and hypertension.

Two studies from areas with high arsenic levels in drinking water in southwestern Taiwan (Chen CJ et al. 1995) and Bangladesh (Rahman et al. 1999) and two studies conducted in areas with low levels of arsenic in drinking water in Wisconsin (Zierold et al. 2004) and central Taiwan (Wang SL et al. 2007) showed consistent associations of arsenic exposure with the prevalence of hypertension. These four studies also showed a consistent dose–response increase in the prevalence of hypertension with increasing arsenic exposure.

Discrepancies in the association between arsenic and the prevalence of hypertension were observed in four studies (Chen Y et al. 2007; Dastgiri et al. 2010; Guo et al. 2007; Yildiz et al. 2008). The study with the strongest association (OR = 16.54; Guo et al. 2007) and the study with the inverse association (OR = 0.71; Yildiz et al. 2008) had small numbers of cases, provided no definition of hypertension, and incorporated no adjustment for relevant confounders. Both studies were highly imprecise with large CIs. The two null studies were large high-quality studies conducted in Bangladesh and the United States (Chen Y et al. 2007; Jones et al. 2011). The study in Bangladesh found no dose–response relationship, despite assessing arsenic at the individual level and defining hypertension based on BP measures (Chen Y et al. 2007). However, this study did find an association between arsenic levels in drinking water with systolic hypertension and pulse pressure levels among participants with low folate and vitamin B intake levels (Chen Y et al. 2007), whereas subgroup analyses by folate and vitamin B concentrations were conducted in the study in the general U.S. population, with no differences (Jones et al. 2011). In the study conducted among the general U.S. population, the association between arsenic exposure and hypertension was not statistically significant, and it was consistent with no association (Jones et al. 2011). However, the magnitude of the association was compatible with a small increased prevalence of hypertension and consistent with the dose–response trend observed in other studies conducted at low-to-moderate exposure levels in Wisconsin and central Taiwan (Jones et al. 2011; Wang SL et al. 2007; Zierold et al. 2004).

The potential association between exposure to inorganic arsenic and the development of hypertension is supported by experimental and mechanistic evidence, especially at high exposure levels. Arsenic promotes inflammation activity, oxidative stress, and endothelial dysfunction through several mechanisms including the activation of stress response transcription factors such as activator protein-1 and nuclear factor-κB (Bunderson et al. 2002; Carmignani et al. 1985; Chen Y et al. 2007; Druwe and Vaillancourt 2010; Pi et al. 2000). *In vitro*, arsenite altered vascular tone in blood vessels by suppressing vasorelaxation (Lee et al. 2003) and increased the expression of cyclooxygenase-2 in endothelial cells (Bunderson et al. 2002; Tsai et al. 2002). In animal models, arsenite increased superoxide accumulation and impaired nitric oxide formation in endothelial cells (Barchowsky et al. 1996, 1999; Lee et al. 2005). Finally, the hypertensive effects of arsenic could be related to the possible chronic kidney effects of arsenic (Chen JW et al. 2011; Hsueh et al. 2009). Additional experimental studies using arsenic exposure levels relevant to human populations are needed to characterize the etiopathogenesis of potential hypertensive effects of arsenic.

## Conclusions

This is the first systematic review and meta-analysis evaluating the relationship between arsenic exposure and hypertension end points. We identified a positive association between elevated arsenic exposure and the prevalence of hypertension, but the implications of this association from a causal perspective are unclear because of the limited number of studies as well as the studies’ cross-sectional design, and methodological limitations. Prospective cohort studies in populations exposed to a wide range of arsenic exposure levels, from low through moderate-to-high levels of exposure, are needed to better characterize the relationship between arsenic and hypertension. Because of the widespread exposure to arsenic worldwide and the high burden of disease caused by hypertension, it is important that high-quality prospective studies are conducted with individual level assessment of arsenic exposure and standardized measurements of BP. The studies should evaluate the shape of the dose response and whether the magnitude of the association is different in susceptible populations, including populations with nutritional deficiencies. If the hypertensive effects of arsenic are confirmed, they could partly explain the association between arsenic and cardiovascular disease (Chen Y et al. 2011; Medrano et al. 2010; Navas-Acien et al. 2005; Smedley and Kinniburgh 2002; DHHS 2005; Wang CH et al. 2007; Wu et al. 1989). Given the widespread arsenic exposure through drinking water and food, even a modest effect of arsenic on hypertension could have a substantial impact on morbidity and mortality (Kwok 2007; Manson et al. 1992).
